# Baculovirus Capsid Display Potentiates OVA Cytotoxic and Innate Immune Responses

**DOI:** 10.1371/journal.pone.0024108

**Published:** 2011-08-30

**Authors:** Paula Molinari, María I. Crespo, María J. Gravisaco, Oscar Taboga, Gabriel Morón

**Affiliations:** 1 Instituto de Biotecnología, Centro Nacional de Investigaciones Agropecuarias (CNIA), INTA Castelar, Buenos Aires, Argentina; 2 Centro de Investigaciones en Bioquímica Clínica e Inmunología (CIBICI-CONICET), Facultad de Ciencias Químicas, Universidad Nacional de Córdoba, Córdoba, Argentina; Federal University of São Paulo, Brazil

## Abstract

Baculoviruses (BV) are DNA viruses that are pathogenic for insects. Although BV infect a range of mammalian cell types, they do not replicate in these cells. Indeed, the potential effects of these insect viruses on the immune responses of mammals are only just beginning to be studied. We show in this paper that a recombinant *Autographa californica* multiple nuclear polyhedrosis virus carrying a fragment of ovalbumin (OVA) on the VP39 capsid protein (BV-OVA) has the capacity to act as an adjuvant and vector of antigens in mice, thereby promoting specific CD4 and cytotoxic T cell responses against OVA. BV also induced *in vivo* maturation of dendritic cells and the production of inflammatory cytokines, thus promoting innate and adaptive immune responses. The OVA-specific response induced by BV-OVA was strong enough to reject a challenge with OVA-expressing melanoma cells (MO5 cells) and effectively prolonged survival of MO5 bearing mice. All these findings, together with the absence of pre-existing immunity to BV in humans and the lack of viral gene expression in mammalian cells, make BV a candidate for vaccination.

## Introduction

The development of vaccines to prevent diseases for which no vaccine currently exists, such as AIDS or malaria, or to treat chronic infections or cancers, as well as the improvement of efficacy and safety of existing vaccines, remains a high priority. In most cases, the development of such vaccines requires strategies capable of stimulating CD8 cytotoxic T lymphocytes (CTLs) and thus, to deliver antigen to MHC class I molecules.

Among other systems, Baculoviruses (BV) have several advantageous features, which make them an attractive new tool for vaccine development. BV are enveloped DNA viruses that infect insects, and require viral transcription factors for propagation. As BV cannot replicate in vertebrate hosts [1], [2], they are considered safe. Their low cytotoxicity, their inability to replicate in mammalian cells and the absence of pre-existing antibodies, make BV candidates for gene therapy, expression vaccines and vector display applications. Furthermore, to the best of our knowledge, there are to date no studies reporting that BV have developed strategies to escape from immune surveillance and thus could hamper immunogenicity, probably because mammals are not their natural hosts.

Recently, BV have become a subject of great interest as immunopotentiators [3]–[7]. Hervas-Stubbs et al. and others have demonstrated that BV have strong adjuvant properties, thereby promoting humoral and CTL responses against co-administered antigens, dendritic cell (DC) maturation and production of inflammatory mediators through mechanisms primarily mediated by IFN-α and β [5].

It has been previously shown that in-frame fusion of foreign sequences to the mature sequence of GP64, an outer glycoprotein of BV, drives the chimeric protein to the surface of the virions [8]. This strategy, known as BV display, has been used to develop recombinant vaccines against foot-and-mouth disease virus (FMDV) [9], *Plasmodium berghei* [10], rubella [11] and bovine herpesvirus-1 (BHV-1) [12] that induced high titers of antigen-specific antibodies.

A transduction strategy, in which the coding sequence of an antigen is driven by the cytomegalovirus (CMV) promoter, was employed by several authors to obtain antigen specific T cell immune responses, resulting in high levels of protection against parasitic diseases [13]–[15]. However, the antigen specific cytotoxicity obtained with this strategy was not very strong [16].

Kukkonen et al. reported the generation of a novel BV displaying a high density of enhanced green fluorescent protein (EGFP) as a fusion to the VP39 capsid protein, while retaining natural infectivity in insect cells [17]. This approach, originally designed to improve the nuclear traffic of BV in mammalian cells, has opened the possibility of performing insertions into the inner capsid of the BV particle. VP39, a 39 KDa polypeptide with monomers arranged in stacked rings around the nucleoprotein core, is the most abundant protein of the nucleocapsid [18]. Also, large polypeptides (up to 28 KDa), instead of single epitopes peptides [19], can be displayed in VP39 by BV, thus allowing a new site for antigens to be delivered by BV vector.

Here, we studied whether BV display using the VP39 capsid protein instead of using the GP64 envelope protein is a valid strategy to induce a T cell immune response. With this objective, we constructed a BV particle expressing the OVA protein on the VP39 capsid protein and assayed its ability to activate adaptive and innate immunity *in vitro* and *in vivo*. Finally, we evaluated if the immune response induced by these BV is strong enough to deal with a tumor challenge. The results showed here clearly demonstrate that BV capsid display, by potentiating T cell immune responses against tumors, could be a promising vaccine vector.

## Results

### BV carrying OVA in the capsid are efficiently internalized by DCs and deliver OVA into the MHC I pathway

**Figure 1:**
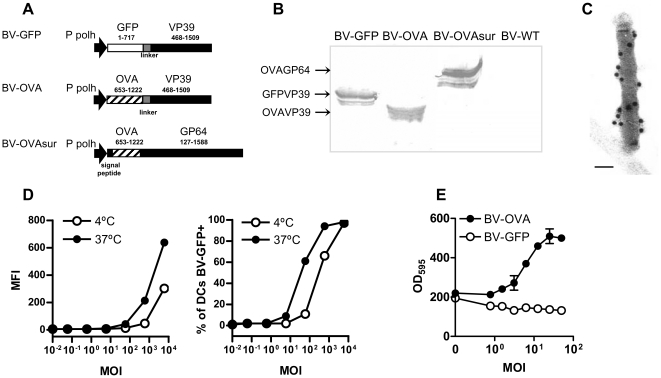
Characterization of BV-OVA. (A) Scheme of Baculovirus (BV) vectors. The Ovalbumin (OVA) (653–1222) and enhanced Green Fluorescent Protein (GFP) (1-717) sequences were cloned 5′ of the VP39 sequence. A linker sequence (GGGGS) was added in the N-terminus of VP39 to provide distance and flexibility for the N-terminal fusion proteins to fold correctly. Production of the fusion protein is driven by the strong polyhedrin promoter. (B) Western blot analysis of purified virions. The virions were purified by ultracentrifugation for 30 min at 131,000× g onto a 25% sucrose cushion. Fusion proteins OVAVP39 (MW 64 KDa), GFPVP39 (MW 67 KDa) and OVAGP64 (MW 89 KDa) were detected with anti-OVA or anti-GFP specific polyclonal antibodies. (C) Immunoelectron microscopy of BV-OVA. Recombinant BV-OVA purified by ultracentrifugation through a 25% sucrose cushion were treated with 1% of Triton 100. Virions capsids were adsorbed to Formvar-coated grids, and the presence and localization of OVAVP39 fusion protein on the capside of the virion was detected with an anti-OVA polyclonal antibody and an anti rabbit IgG-gold conjugate. The figure is representative of all fields examined. Bar = 100 nm. (D) Uptake of BV by Bone Marrow-derived Dendritic Cells (BMDCs). The BMDCs were incubated with BV-GFP for 2 hours at 37°C or 4°C. Then, cells were washed, stained with anti-CD11c and analyzed on a FACSCanto II. In all cases, a minimum of 2×10^5^ events was acquired. Results are representative of two independent experiments and are expressed as the geometric mean of the fluorescence intensity (MFI) of the FL1 channel in total DCs or the percentage of DCs which are positive for FL1 channel. (E) *In vitro* OVA presentation by DCs infected with BV-OVA. Spleen CD11c+ cells (2×10^5^) were incubated *in vitro* with BV-OVA (•) or BV-GFP (○), and cultured overnight with 10^5^ B3Z cells/well. Then, cells were washed and the presentation of the OVA_257-264_ epitope to B3Z cells was monitored by the activity of β–galactosidase with a colorimetric assay. Results are representative of two independent experiments and are expressed as mean +/− SEM of the optical density at 595 nm (OD_595_, n = 3).

To maximize the possibility of obtaining functional fusion proteins and capsid assembly, we constructed a transfer plasmid that enabled the fusion of a truncated sequence of OVA or the entire sequence of EGFP to the N-terminus of a second copy of VP39 under the regulation of the strong polyhedrin promoter (BV-OVA and BV-GFP, respectively) (Fig. 1A). Purified virus preparations from Sf9 cells infected with BV-GFP and BV-OVA produced the predicted 67 KDa and 62 KDa protein bands respectively, as determined by Western blot (Fig. 1B). However, no bands were detected in cells infected with non-recombinant BV (BV-WT), indicating the incorporation of EGFP and OVA as a part of the virus structure. To confirm that fusion proteins were incorporated on the viral capsid, purified BV-OVA was analyzed by immunogold labeling for electron microscopy using an anti-OVA antibody. The viral capsid showed a typical rod-shaped morphology and their surfaces were gold labeled (Fig. 1C). Immunoblotting and flow cytometry revealed that about 1170 OVA molecules were incorporated per virus particle (data not shown).

Dendritic cells (DCs) are professional antigen presenting cells and the preferred target cells of vaccine vectors. To evaluate the ability of BV as carriers of antigen, bone marrow-derived dendritic cells (BMDCs) were incubated with BV-GFP at 37°C for 2 hours at different multiplicities of infection (MOI) and BV uptake was analyzed by flow cytometry. We observed an efficient uptake of BV-GFP by BMDCs that increased with MOI (Fig. 1D). The same experiment conducted at 4°C (as negative control for internalization) rendered a lower binding of BV to DCs. We then examined whether DCs can process antigens carried by BVs, using a classical *in vitro* antigen presentation assay. Splenic DCs incubated with BV-OVA were cultured with B3Z cells, a CD8 T cell hybridoma which recognizes the peptide corresponding to aminoacids 256 to 264 in OVA (OVA_256-264_) associated to H2-K^b^. We observed that DCs incubated with BV-OVA activated B3Z cells, whereas DCs incubated with control BV-GFP did not stimulate B3Z cells (Fig. 1E), showing that BV-OVA had the capacity to deliver antigens into the MHC I pathway. Taken together, these results demonstrated that BV can carry heterologous antigens in the capsid, be internalized by DCs, and access the MHC I pathway for presentation to CD8 T cells.

### BV induce DC maturation and CD8 T cell activation

**Figure 2:**
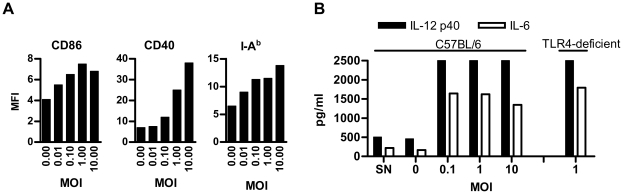
BV induce BMDCs maturation. (A) Maturation of BMDCs by BV. BMDCs were incubated for 18 hours with BV-WT or mock. Then, cells were stained with anti-CD11c and one of the following antibodies: anti-CD40, CD86 or I-A^b^, and analyzed on a FACSCanto II. In all cases, a minimum of 2×10^5^ events was acquired. Results are representative of two independent experiments and are expressed as the geometric mean of the fluorescence intensity (MFI) for each indicated molecule in total DCs. (B) Production of inflammatory cytokines. BMDCs from C57BL/10 ScCr (TLR4 deficients) and C57BL/6 (TLR4 competents) were incubated for 18 hours with BV-WT, or a supernatant from mock infected Sf9 cells (SN), and then levels of IL-6 and IL-12 p40 were determined in supernatants of BMDCs by ELISA. Results are representative of two independent experiments.

We investigated if BV were able to induce maturation of DCs. BMDCs incubated with BV-WT for 18 hours showed by flow cytometry analysis an increase in the expression of the phenotypic activation marker CD40 and, to a lesser extent, of CD86 and MHC II (Fig. 2A), as well as in the production of the inflammatory cytokines IL-6 and IL-12 p40 (Fig. 2B), as determined by ELISA in culture supernatants. Similar results were obtained with BMDCs from TLR4 deficient mice, indicating that the maturation observed in TLR4 competent DCs was due to BV and not to LPS contamination within BV formulations (Fig. 2B). DCs exposed to supernatants from mock infected Sf9 cells (SN) did not exhibit either IL-6 or IL-12p40 secretion (Fig. 2B), showing that no other constituent from the insect cell cultures than BV were responsible for DC stimulation. These results clearly show that BV are efficiently internalized by DCs, which then mature and secrete proinflammatory cytokines.

**Figure 3:**
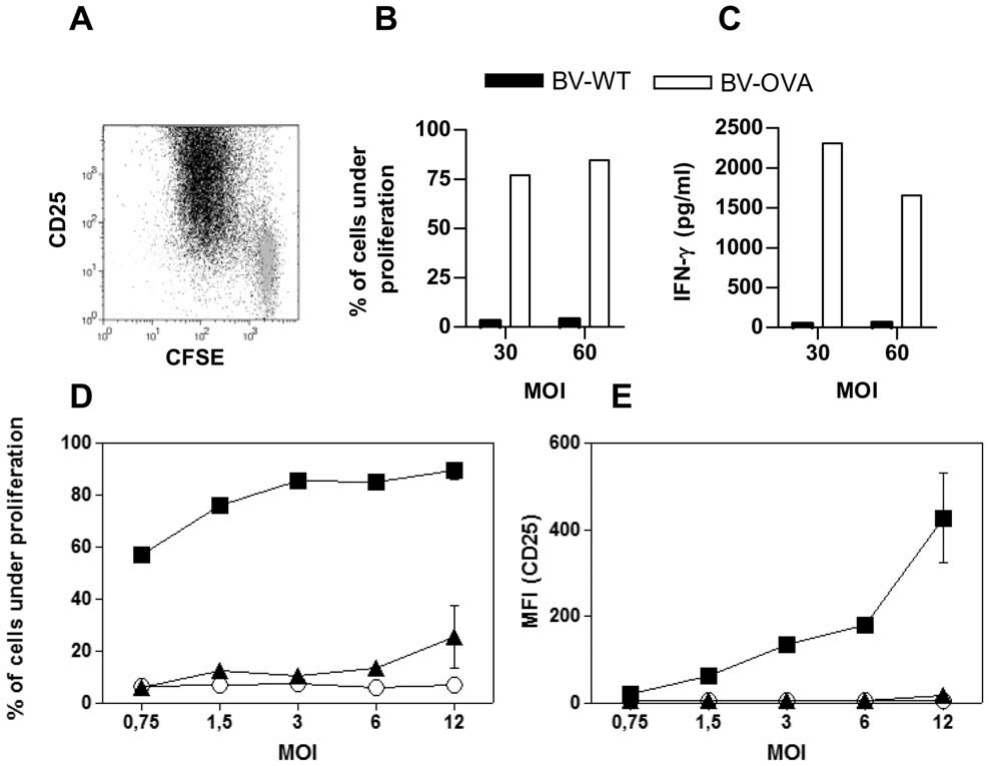
BV-OVA induces CD8 T cell activation. Splenic CD11c+ cells (2×10^5^) purified from C57BL/6 mice were incubated with BV-WT, BV-OVA or BV-OVAsur at the indicated multiplicity of infection (MOI) for 120 minutes and washed twice. Then, DCs were cultured for 3 days with CFSE-labeled CD8 T cells from OT-I mice (having a transgenic T cell receptor which recognizes OVA_257-264_ in the context of H-2K^b^). T cell proliferation and CD25 expression were analyzed by flow cytometry, after labeling cells with an anti-CD3 antibody and exclusion of dead cells with 7AAD. (A) A representative dot plot overlay of cell proliferation vs CD25 expression of CD8 T cells cultured with DCs, which were preincubated with BV-OVA (black dots) or BV-WT (gray dots) at a MOI of 30. (B) Percentage of CD8 T cells undergoing one or more rounds of proliferation and (C) IFN-γ content, assessed by ELISA, in supernatants of culture of CD8 T cells with DCs preincubated with BV-OVA or BV-WT. (D) T cell proliferation and (E) CD25 expression of CD8 T cells co-cultured with DCs preincubated with BV-OVA (▪), BV-OVAsur (▴) or BV-WT (○). Results are representative of at least two independent experiments and are expressed as mean +/− SEM (n = 4).

As BV-OVA was able to deliver OVA to the MHC I pathway, we evaluated its ability to induce CD8 T cell activation. With this purpose, splenic DCs were first incubated with BV-OVA and then co-cultured with CD8 T cells from OT-I mice. Activation of CD8 T cells was assessed by a carboxyfluorescein diacetate, succinimidyl ester (CFSE)-dilution proliferation test as well as by CD25 expression and IFN-γ secretion by T cells. Incubation of DCs with BV-OVA led to strong proliferation (Fig. 3A and B) and differentiation of naïve OT-I CD8 T cells into effector cells, as determined by CD25 expression (Fig. 3A) and IFN-γ production (Fig. 3C). In contrast, a BV formulation displaying an OVA fragment on the BV envelope by fusion to its GP64 surface protein (BV-OVAsur, Fig. 1A, B) was unable to activate either CD8 T cell proliferation (Fig. 3D) or CD25 upregulation (Fig. 3E). Collectively, these findings indicate that BV capsid display is a very efficient strategy for delivering antigens into the MHC I pathway in DCs and for activating naïve CD8 T cells.

### BV capsid display elicits an OVA-specific CTL response

**Figure 4:**
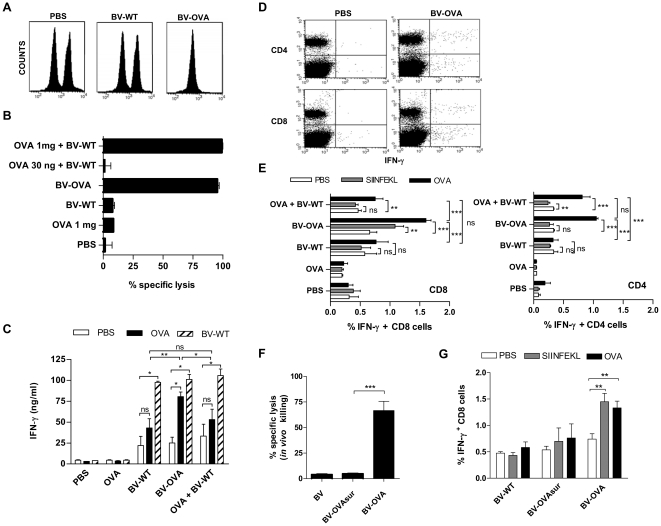
Anti-OVA CTL response in mice injected with BV-OVA. C57BL/6 mice were immunized by a single i.v. injection of 5×10^7^ PFU BV-WT, BV-OVA, BV-OVAsur, BV-WT + OVA (30 ng or 1 mg), OVA alone (1 mg) or PBS. Seven days later, immunized mice received an i.v. injection of a mixture (1∶1) of OVA_256-264_ peptide-loaded CFSE^high^ and unloaded CFSE^low^ splenocytes as target cells. (A) A representative histogram of remaining CFSE^high^ and CFSE^low^ cells in control and BV-OVA immunized mice 20 hours after injection of target cells is shown. (B) Percentage of specific *in vivo* killing of one representative experiment. (C) IFN-γ content in supernatants of spleen cells from immunized mice determined by ELISA. Spleen cells were recovered and cultured for 48 hours in the presence of OVA protein or BV-WT. As control, spleen cells without stimulus were also cultured. *, p<0.05; **, p<0.01. (D, E) IFN-γ intracellular staining on splenocytes from immunized mice with 30 ng OVA+BV-WT, BV-WT, BV-OVA, OVA alone or PBS, as indicated above. Spleen cells were recovered and cultured for 12 hours in the presence of OVA protein or OVA_256-264_, and incubated in the presence of brefeldin A for 6 additional hours. Then, cells were labeled for CD4 or CD8 markers and intracellular IFN-γ. (D) A representative dot plot is shown. (E) Percentage of IFN-γ+ CD4 or CD8 T cells. **, p<0.01; ***, p<0.001. (F) Comparison of specific *in vivo* killing between mice immunized with BV-OVA vs BV-OVAsur. ***, p<0.001. (G) Comparison of the frequency of IFN-γ-producing CD8 T cells in splenocytes from BV-OVA vs BV-OVAsur immunized mice. **, p<0.01. Results are representative of at least two independent experiments and are expressed as mean +/− SEM (n = 4).

The next step was to evaluate whether the activation of naïve CD8 T cells by recombinant BV led to the induction of an adaptive CTL response against the heterologous antigen. Seven days after a single i.v. injection, BV-OVA induced a vigorous CTL response specific to the OVA_257-264_ epitope (Fig. 4A, B), as determined by an *in vivo* killing assay using CFSE-labeled target cells. One mg of OVA protein coadministered with BV elicited an equivalent CTL response to that induced by BV-OVA, whereas 30 ng of OVA (the same amount as that of the OVA contained in the BV-OVA formulation) coadministered with BV did not elicit a detectable CTL response (Fig. 4B). Thus, our results show that although BV have a good adjuvant capacity to elicit CTL responses against soluble antigens, they are far more efficient when antigen is carried by VP39 capsid protein, as they allow to hugely reduce the amount of required antigen for the same response.

The CTL response was accompanied by a high production of IFN-γ upon *in vitro* restimulation of immune spleen cells with OVA protein (Fig. 4C). Furthermore, when spleen cells from mice immunized with BV-OVA, or with BV-WT alone or combined with OVA protein were restimulated with BV-WT, we observed high and similar levels of IFN-γ (Fig. 4C). This showed that BV elicited a strong T cell response not only to OVA but also against BV antigens, produced by CD4 and CD8 T cells (data not shown). We also observed that in the absence of restimulation, the supernatants of all experimental groups immunized with any of the BV formulations had a higher IFN-γ content than mice injected with OVA alone or PBS. The same result was observed in supernantants of mice immunized with BV-WT and restimulated with OVA. These last observations could be attributed to the strong anti-BV T cell response, which does not need further restimulation to be detected.

In order to identify the source of IFNγ in spleen cells and evaluate the quality of T cell response, we performed an intracellular IFN-γ staining of splenocytes from all experimental groups. As determined by flow cytometry, immunization with BV-OVA elicited the production of IFN-γ by CD4 and CD8 T cells (Fig. 4D), whereas the injection of OVA protein (30 ng) in combination with BV-WT elicited OVA-specific IFN-γ production only in CD4 T cells (Fig. 4E), strongly indicating that to obtain sufficient and efficient CD8 T cell activation, antigen must be contained in the BV capsid. As for ELISA bulk determinations, all experimental groups immunized with any of the BV formulations had a higher frequency of IFN-γ-producing CD4 and CD8 T cells than the same cells of mice injected with OVA alone or PBS in the absence of the specific stimulus, reinforcing the observation that BV induce such a strong T cell response that it does not need further restimulation to be detected.

Next, we compared the ability of BV-OVAsur and BV-OVA (containing a similar number of OVA insertions in both preparations) to induce a CTL response. Seven days after immunization, mice injected with BV-OVAsur failed to elicit a CTL response (Fig. 4F) and no OVA-specific IFN-γ secreting CD8 T cells were detected after *in vitro* restimulation of spleen cells with OVA (Fig. 4G). When the same comparison was performed by employing a BV-OVAsur containing around 5 times more OVA insertions than BV-OVA, BV-OVAsur was able to induce a mild anti-OVA CTL response, much weaker than the response induced by BV-OVA (approximately 55% of specific lysis vs around 95%, respectively, data not shown). These results showed that BV capsid display is a much more efficient strategy to activate CD8 T cell mediated responses than BV envelope display.

**Figure 5:**
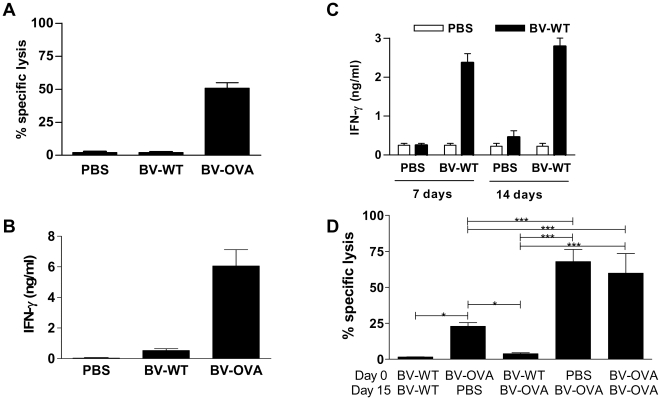
Anti-OVA CTL response in mice injected with BV-OVA is long-lasting and not affected by repeated immunization with the same vector. (A, B) C57BL/6 mice were immunized by a single i.v. injection of 5×10^7^ PFU BV-WT, BV-OVA or PBS. One hundred and ten days later, immunized mice received an i.v. injection of a mixture (1∶1) of OVA_256-264_ peptide-loaded CFSE^high^ and unloaded CFSE^low^ splenocytes. Twenty hours later, spleen cells were recovered to determine (A) the percentage of specific *in vivo* killing by flow cytometry and (B) IFN-γ content by ELISA in supernatants of spleen cells from immunized mice, cultured for 48 hours in the presence of OVA protein. (C) C57BL/6 mice were immunized by a single i.v. injection of 5×10^7^ PFU BV-WT or PBS. Seven and fourteen days later, spleen cells were recovered and cultured with BV-WT (MOI 5) for 48 hours. The IFN-γ content in supernatants was determined by ELISA (D) C57BL/6 mice were immunized by a single i.v. injection of 5×10^7^ PFU BV-WT, BV-OVA or PBS. Fifteen days later, mice received a second i.v. injection of 5×10^7^ PFU BV-WT, BV-OVA or PBS, combined as shown in the figure. Seven days later, immunized mice received an i.v. injection of a mixture (1∶1) of OVA_256-264_ peptide-loaded CFSE^high^ and unloaded CFSE^low^ splenocytes. Twenty hours later, spleen cells were recovered and the percentage of specific *in vivo* killing was determined by flow cytometry. *, p<0.05; ***, p<0.001. Results are representative of at least two independent experiments and are expressed as mean +/− SEM (n = 5).

The anti-OVA CTL response remained evident at least 110 days after a single injection of BV-OVA (Fig. 5A), without the need of a further boost. Moreover, spleen cells recovered from the same mice still produced significant amounts of IFN-γ upon restimulation with OVA (Fig. 5B). This latter result reaffirmed the efficiency of single doses of BV, which elicited a strong and long-lasting CD8 T cell response to an heterologous antigen.

Strong B and T cell immunogenicity against vectors can limit the T cell response against the foreign antigens they carry [20]. Although a single injection with BV-OVA is enough to obtain a long lasting OVA-specific CTL response without further boosting, the strong anti-BV T cell response (which remains very high at least 14 days after a single injection, Fig. 5C) may be able to constrain repeated injections of BV to boost the immune response. Thus, in order to evaluate this possibility, we measured the OVA-specific CTL response in mice injected twice with BV-OVA (on days 0 and 15). As showed in Figure 5D, anti-OVA CTL response on day 21 after a single BV-OVA injection (Group BV-OVA/PBS) was reduced compared to CTL response 7 days after immunization (Group PBS/BV-OVA). However, mice receiving two repeated doses of BV-OVA had an equivalent CTL response against OVA (Group BV-OVA/BV-OVA) than mice immunized with a single BV-OVA injection on day 15 (Group PBS/BV-OVA). Conversely, previous injection with BV-WT abrogated the induction of anti-OVA CTL upon immunization with BV-OVA (Group BV-WT/BV-OVA), showing that BV-immune mice had an impaired ability to mount *de novo* anti-OVA responses. In this way, a strong anti-BV immune response prevents the induction of a primary response, but does not constrain the maintenance and boosting of an already established anti-OVA CTL response after repeated immunizations.

### BV induce an innate immune response

**Figure 6:**
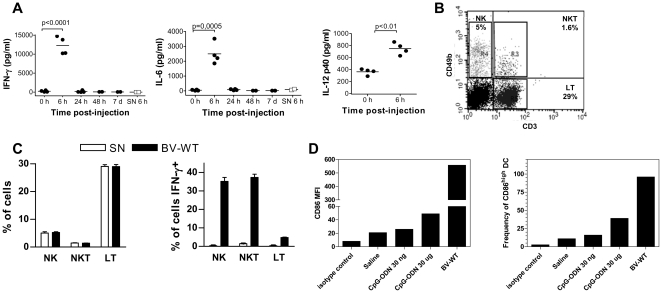
Innate immune response in mice injected with BV. (A) Induction of inflammatory cytokines by BV. C57BL/6 mice were i.v. injected with 5×10^7^ PFU BV (•) or with supernatants of uninfected insect cells (SN, □), and at the times shown sera were collected and assayed for IL-6, IFN-γ and IL12p40 by ELISA. Sera collected before the injection were employed as control. Results are representative of two independent experiments. The line depicted in each group corresponds to the mean of each group of dots. (B, C) Activation of NK, NKT and T cells by BV. C57BL/6 mice were i.v. injected with 5×10^7^ PFU BV, and 3 hours post injection splenic populations were incubated in the presence of BFA for an additional 6 hours. Then, cells were labeled for CD49b and CD3 markers and intracellular IFN-γ. (B) A representative dot plot of the proportion of NK (CD49b+ CD3-), NKT (CD49b+ CD3+) and T (CD49b- CD3+) cells in the spleen of a control mouse and (C) the percentage of NK, NKT and T cells (left) and proportion of IFN-γ+ NK, NKT and T cells after BV injection (right) are shown. Results are representative of two independent experiments and are expressed as mean +/− SEM (n = 4). (D) *In vivo* maturation of DCs by BV. C57BL/6 mice were injected i.v. with saline, 5×10^7^ PFU BV-WT and 30 ng or 30 µg CpG-containing oligodeoxinucleotide (CpG-ODN) 1826. Eighteen hours later, their CD11c+ spleen cells were labeled with anti-CD86 and analyzed on a FACSCanto II flow cytometer. In all cases, a minimum of 2×10^5^ events was acquired. Results are representative of two independent experiments and are expressed as the geometric mean of the fluorescence intensity (MFI) and as the frequency of CD86+ cells in total DCs. Results are representative of at least two independent experiments.

In order to evaluate the stimulation of an innate immune response by the BV, we studied the production of IFN-γ, IL-6 and IL-12p40 in C57BL/6 mice shortly after an i.v. injection of BV-WT or SN. High levels of IFN-γ, IL-6 and IL-12p40 were detected in the sera of BV-WT injected mice 6 hours post injection, which then returned to basal levels after 24 hours (Fig. 6A). However, none of these cytokines were detected in sera of mice injected with SN. Interferon-γ producing cells in spleen were identified as NK and NKT cells (with 36 and 38%, respectively, of these cells being IFN-γ+) (Fig. 6B, C). Related to this, no changes in the proportion of these cell populations were observed in spleen after BV injection.

Intravenous BV injection also stimulated *in vivo* DC maturation, as shown by up-regulation of CD86 (Fig. 6D) and MHC II and CD40 molecules (data not shown). The BV genome contains bioactive CpG motifs whose frequency is similar to that observed for *Escherichia coli* and herpes simplex virus DNAs and significantly higher than that in murine and entomopoxvirus DNAs [3]. TLR9 is essential for the immune response to CpG-rich DNA and it is considered to be the most probable receptor involved in DC activation by BV [3], [21]. In order to evaluate the extent to which DC maturation is induced by BV, we also measured the expression of CD86 on DCs from mice injected i.v. with 30 ng or 30 µg of CpG-containing oligodeoxynucleotide (CpG-ODN) 1826. BV DNA contains at least 7 CpG different motifs, which in total represent a total count of 344 copies of CpG motifs in the entire BV genome (Table 1). Therefore, in a regular dose of 5×10^7^ plaque forming units (PFU) BV-WT there are at least 2.9×10^−5^ nmol CpG motifs, which is approximately 100,000 times lower than the amount of CpG motifs contained in 30 µg CpG-ODN 1826 (5.5 nmol). The up regulation of CD86 expression in mice injected with 30 µg CpG-ODN complexed with DOTAP was notably lower than that observed after 5×10^7^ PFU BV injection and was almost not noticeable in mice injected with 30 ng CpG-ODN, clearly indicating that BV is a much more efficient inducer of DC maturation.

**Table 1:** Estimation of the number of CpG motifs in AcNMPV genome.

CpG motif	# of copies
TGACGTT	11 copies
GACGTT	37 copies
CACGTT	119 copies
AACGTC	40 copies
AGCGTC	35 copies
GGCGTC	Not present
GGCGTT	56 copies
AGCGTT	46 copies

The frequency at which each CpG hexamer appeared in the BV genome was determined by using the GenBank accession number for the complete genome of AcNPV NC 001623.

Taken together, our results demonstrated that BV promote innate immune responses by inducing inflammatory cytokines, IFN-γ production by NK and NKT cells and *in vivo* DC maturation.

### BV-capsid display strongly potentiates antitumor immune response

**Figure 7:**
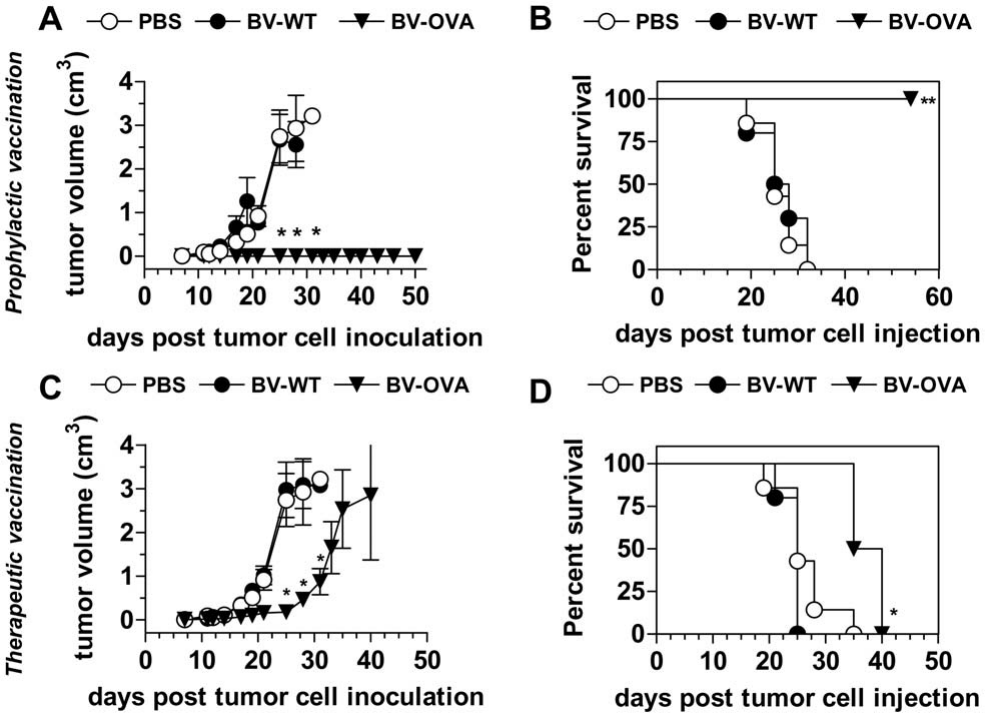
Prophylactic and therapeutic vaccinations with BV-OVA. (A, B) Prophylactic vaccination. Groups of eight C57BL/6 mice were immunized by a single i.v. injection of 5×10^7^ PFU BV-WT, BV-OVA or PBS. Seven days later, immunized mice were challenged with a s.c. injection of 1×10^5^ syngenic OVA-expressing melanoma cells (MO5 cells). (A) Progression of tumor volumes and (B) survival are shown. (C, D) Therapeutic vaccination. Groups of eight C57BL/6 mice were challenged with an s.c. injection of 1×10^5^ MO5 cells. Seven days later, mice showing palpable tumors were treated with 5×10^7^ PFU BV-WT, BV-OVA or PBS by i.v. injection. At days 11 and 17 post-tumor cell inoculation, mice were s.c. injected near to the tumors with 1×10^7^ PFU BV-WT, BV-OVA or PBS respectively. At day 21 post-tumor cell inoculation, mice were intratumorally injected with 1×10^7^ PFU BV-WT, BV-OVA or PBS respectively. (C) Progression of tumor volumes and (D) survival are shown. Results are representative of three independent experiments and are expressed as mean+/− SEM (n = 8). *, p<0.05; **, p<0.01.

In order to test the efficacy of BV vaccination we investigated whether the immune response induced by BV-OVA was strong enough to prevent tumor implantation and expansion. First, we conducted a classical prophylactic vaccination scheme. Mice were immunized with BV-OVA and 7 days later challenged with a subcutaneous (s.c.) injection of MO5 cells. All mice vaccinated with BV-OVA survived without developing tumors whereas mice vaccinated with BV-WT or PBS succumbed (Fig. 7A, B). Moreover, all mice vaccinated with BV-OVA were free of tumors until the end of the experiment (more than 60 days). To verify that the protection provided by BV-OVA was specific to the OVA antigen expressed by MO5 cells, we challenged groups of vaccinated mice with the untransfected parental cell line B16. In these experiments, tumors grew in all groups at similar rates (data not shown), indicating that the anti-tumoral immune response was mediated by a specific anti-OVA immune response induced by BV-OVA. Furthermore, mice vaccinated with BV-OVA and challenged 7 days later with MO5 cells became protected from a second challenge at day 120 after immunization with B16 cells (data not shown), indicating that the prophylactic response elicited by BV-OVA and the subsequent challenge with MO5 cells triggered the development of a long lasting anti-B16 cell specific response.

To examine further the potential of the BV capsid display, we conducted a therapeutic vaccination scheme. Mice were injected with a s.c. dose of MO5 cells 7 days before being immunized with BV-WT, BV-OVA or PBS and subsequently injected by near tumor s.c. or intratumoral injections on days 11, 17 and 21 post-tumor cell inoculation, in order to maintain a state of continuous inflammation around the site of tumor implantation and consequently increase mice survival. Tumors grew much faster in mice injected with PBS or BV-WT than in mice injected with BV-OVA. Survival was 100% in mice injected with BV-OVA on day 32 post-tumor injection, with all mice died by day 42. In PBS or BV-WT immunized mice only 50% were alive on day 28 and all of them were dead by day 32 (Fig. 7C, D). Therefore, a significant prolongation in survival was found for BV-OVA mice compared with PBS or BV-WT ones.

These results demonstrate that recombinant BV is sufficient to establish a protective immunity against MO5 challenge, thus showing the potential of BV capsid display as a new strategy of vaccination.

## Discussion

The baculovirus system has been previously shown to be capable of displaying a foreign protein on the virion surface [9]–[12], [22], usually by using GP64, its major surface glycoprotein. It has been reported that *in vivo*, after i.v. injection, BV are taken up by the liver and spleen, and preferentially infect DCs and B cells in the spleen [6]. BV can reach the cytoplasm of mammalian cells by a mechanism similar to the one used in insect cells [17], [23]–[25], with this process starting with endocytosis, being followed by acid-induced fusion of the virus envelope to the endosome (probably mediated by GP64) and by escape of the virus capsid to the cytosol [26]. Therefore, recombinant GP64 should remain on the luminal side of the endosome membrane. Under these circumstances, it seems unlikely that the antigen displayed on the BV envelope would be able to efficiently reach the cytoplasm and consequently would preferentially trigger CD4 T cells. As foreseen by this hypothesis, antigen displayed on GP64 failed to produce a good CD8 T cell response, but did induce an effective CD4 T and B cell ones [16]. In contrast, antigen contained in the capsid should be able to reach the cytosol and preferentially trigger CD8 T cells. So far, to our current knowledge there have been no reports about antigen display on VP39 capsid protein. Thus, we constructed a BV vector bearing OVA in VP39, based on the hypothesis that in this way OVA could enter into the MHC I pathway. Consequently, the immunopotentiation properties and the antigen delivery ability of BV would be combined in one single vector. Consistent with this assumption, BV-OVA showed the capacity to deliver OVA to the MHC I pathway, thereby activating naïve CD8 and inducing an OVA-specific cytotoxic response. In contrast, recombinant BV displaying OVA in GP64 (BV-OVAsur) failed to induce a cytotoxic response and IFNγ-secreting CD8 T cells. Furthermore, BV-OVAsur could not activate CD8 T cell proliferation in an *in vitro* assay. All these data clearly establish the convenience of capsid display over envelope display for CTL triggering. In agreement with our results, Strauss et al. showed that BV containing the *Plasmodium falciparum* circumsporozooite (CS) protein coding sequence, under the control of the CMV promoter, allowed transduction and the novo synthesis of CS expression in the cytosol of human DCs, thus enabling induction of CD8 T cell responses [14]. On the other hand, display of CS in the baculovirus envelope by fusion to GP64 induced a poor activation of CD8 T cells [14].

We have not yet elucidated the mechanism by which the OVA displayed on the BV capsid is delivered to the MHC I pathway. Nevertheless, as proposed above, it is possible that whole BV capsid reaches the cytoplasm of DCs after a fusion event between the viral envelope and the endosome membrane, thus generating MHC I binding peptides by proteasome digestion of the entire capsid. Another possibility is that BV viral particles experience controlled endosomal degradation before reaching the cytoplasm for proteasome processing. In fact, both alternatives may be taking place, as BV-OVA also activate OVA-specific IFNγ-secreting CD4 T cells. Supporting this hypothesis we have observed in preliminary studies using electron microscopy that recombinant BV-OVA capsids can be found in the cytoplasm of BMDCs (data not shown).

Although it has been shown that BV have adjuvant properties when they are co-administered with particulate structures such as latex beads or virus-like particles (VLPs) [5] or tumor cells [7], in the present study we have observed that the delivery of antigen and adjuvant in the same BV particle rendered a stronger CTL response. Indeed, when soluble OVA was coadministered with BV-WT, it was necessary to immunize with 30 times more OVA than the quantity of OVA displayed by BV-OVA to attain the cytotoxic response achieved by BV-OVA. This result would suggests that a differential pathway of antigen processing could have taken place between both forms of OVA administration in DCs, with one being more efficient for BV-OVA by direct access to cytosol and other less efficient, requiring endosomal traffic for soluble OVA coadministered with BV.

BV injection also induces a T cell immune response against BV themselves, regardless the type of BV form, as shown by a high secretion of IFN-γ by CD4 and CD8 T cells. Strauss et al. [14] also observed that splenocytes harvested from baculovirus-injected mice routinely had a high frequency IFN- γ-producing T cells. One single injection of BV-OVA was sufficient to induce an OVA-specific CTL response, but more than one injection was necessary for anti-tumor therapeutic vaccination. Interference between responses to individual epitopes presented by MHC class I molecules results in the well-established phenomenon of immunodominance in multispecific CD8 T-cell responses, with this immunodominance limiting the priming of responses with extensive repertoire diversity [27]–[30]. Also, pre-existing antibodies against the vector can limit the induction of immune response against the inserted antigen (unpublished results). Therefore, a strong immune response against BV might constrain the boosting of the T-cell response against the foreign OVA inserted in BV. Effectively, mice immunized with BV-WT cannot generate anti-OVA CTL responses after immunization with BV-OVA (Figure 5D). However, two homologous injections with BV-OVA did not affect the quality of the anti-OVA CTL response. More importantly, this CD8 T-cell response which decreased after the peak on day 7 after initial priming was boosted with a second immunization without *de novo* synthesis of antigen. Similar results were reported by de Mare et al. [31]. Using recombinant Semliki Forest virus (rSFV) expressing E6E7 antigen from human papillomavirus, they demonstrated that secondary immune responses against E6E7 are neither affected by vector-specific antibodies nor by CTL-mediated killing of infected cells. Instead, the presence of the antigen during the prime immunization appeared to be the main determinant for the boosting efficacy. However, both quantitative and qualitative differences in the CD8 T cell response generated by different viral vectors can be found using homologous prime-boost regimens: homologous immunization with SFV provided higher tumor protection than homologous immunization with recombinant adenovirus [32].

We show here that BV are internalized by DCs and induce their maturation and the production of the pro-inflammatory cytokines IL-6 and IL-12. Similar results were obtained in other studies using murine and human DCs [14], [33]. Hervas-Stubbs et al. showed that BV induced phenotypic maturation markers *in vivo* in conventional and plasmacytoid DCs (cDC and pDC, respectively) [5], apparently through the action of the BV DNA on DCs. However, the mechanisms participating in DC activation by BV are still not fully understood. Hervas et al. [5] reported that BV DNA inactivation abrogates *in vivo* DC maturation and CTL response in mice, suggesting that the adjuvancy of BV could be based on the recognition of their DNA content rather than on other molecules acting in BV such as GP64 [21]. Here we have found that BV are much more effective than CpG-ODNs, other DNA-like immunostimulators which bind TLR9. Indeed, an injection of BV containing a 10^5^ times lower content of CpG motifs than a regular dose of CpG-ODN 1826 induces *in vivo* a much higher up-regulation of CD86 in splenic DCs, revealing the potential of combining a antigen delivery vector with an immunostimulant. Related to this, type I IFN seems to be the primary intermediary involved, although mechanisms independent of type I IFN signaling are also implicated [5]. BV are capable of inducing the production of type I IFNs in splenic DCs through a partially MyD88/TLR9-independent pathway [3], in which cytoplasmic RIG-1 and MDA-5 RNA receptors do not participate [21]. In humans, only B cells and pDCs express TLR9 [34]–[36]. However, there is cumulative evidence that shows that although human cDCs do not express TLR9, pDCs can be a key cell for BV responses through two different, perhaps confluent ways. First, pDCs can help cDCs to present antigens carried by BV by, for example, type I IFN secreted by pDCs stimulated with BV [37], [38] or alternatively by Il-15, essential for CpG-induced immune activation [39]. Second, pDCs could directly process and present antigens carried by BV to CD8 T cells [40]–[42]. It is still not clear whether, in a vaccinal study, pDCs have a significant role, neither whether BVs can enter into human pDCs or any other human DC and activate type I IFN through TLR9 ligation. However, the current evidence supports a potential role for pDCs in induction of immunity by BV.

The efficacy of the strong CTL and innate immune response elicited by BV was examined by the capacity of BV-OVA to confer protection against the classical MO5 melanoma tumor model. This model, although not relevant to any natural occurring tumor, has been extensively employed to test the efficacy of vaccination protocols. BV induced a 100% protection against MO5 murine melanoma cells in immunized mice under a prophylactic scheme of immunization. Moreover, when BV-OVA was used as a therapeutic vaccine, a decline in tumor growth speed and a prolongation in survival were observed, demonstrating that the acquired immune response induced by BV is strong enough to eliminate tumor cells. Kitajima et al. [6] reported that injection of BV one day after tumor cell inoculation induced a NK cell-dependent antitumor immunity. More recently, Suzuki et al. showed that DCs pre-cultured with BV were able to activate NK, thus demonstrating an indirect means of activation [33]. In the present study, we have reported that BV injection induced high levels of IFN-γ soon after immunization, which were principally secreted by NK and NKT cells. Although we did not conduct experiments to evaluate the participation of NK cells in the anti-tumor immune response induced by BV-OVA, T cells rather than NK cells seem to be the main cytotoxic cells involved in tumor cell destruction, as the parameters of tumor growth and survival in BV-WT-injected mice were quite similar to those observed in PBS-injected mice. NK and NKT cells may cooperate with DCs to establish the CTL response elicited by BV. There is evidence showing that NK and NKT cells maintain bidirectional interactions with DCs [43]. Release of IFN-γ by NKT cells induces DC maturation [44] and the DC maturation induced by TLR stimuli is enhanced by NKT cells [45], [46]. Thus, it is possible that NKT cell interactions with DCs early after BV injection could contribute to the outcome of the immune response against an antigen carried by BV, a very interesting hypothesis that remains to be investigated.

It is generally accepted that CD8 T cells, with the ability to directly lyse tumour cells and to secrete interferon IFN-γ and TNFα, are important T cell components of the adaptive tumoral response [47], [48]. Sometimes, this CD8 T cell response is triggered independently of the presence of a tumor specific CD4 T cell response [49] whereas in other cases the CD4 and CD8 T cells can act separately in tumor rejection as in the case of VLPs [50]. BV induce the development of IFN-γ-secreting OVA-specific CD4 and CD8 T cell responses and the presence of a cytotoxic response. However, we have not yet determined which of these cell populations are mediating tumor eradication nor the mechanisms involved in the prophylactic and therapeutic settings. Nevertheless, the strong secretion of IFN-γ could be one important mediator, as IFN-γ has been reported to have a direct antiproliferative effect on the melanoma cells and to be the most relevant mediator in tumor eradication rather than perforin and TNFα in a model of vaccination with an adenoviral vector [49].

Among the enlarging body of candidate vectors for vaccination, which have the capacity to extend survival in mice bearing tumors or even eradicate them when employed in combination with other strategies [51], BV capsid display system has several additional and potential advantages as a vaccine. The use of purified virions as immunogens alleviates the need for additional adjuvants to the vaccine formulation due to the intrinsic immunostimulatory effect of BV. Furthermore, BV can display large polypeptides in VP39, which are able to hold multiple epitopes, thereby rendering BV suitable for a broad diversity of MHC haplotypes and consequently for potential use in humans and animals. The combination of several BV carrying different tumoral antigenic proteins, with each being applied at different times could also be a strategy to limit the immune editing that cancer cells carry out under the selective pressure of a strong immune response [52]. A significant advantage of BV is that, up to now, they have revealed being unable to replicate in mammals and therefore do not need any inactivation or attenuation procedures. We have found that the injection of mice with BV did not lead to the release of hepatic enzymes to blood or to any visible (with light microscopy) alteration of the hepatic histology at least up to day 7 post BV-injection (data not shown), suggesting that BV represent a safe alternative. Finally, another attractive feature of this vector system is that it is possible to produce large amounts of BV in serum-free medium at low cost.

Summing up, its ability to impact on the innate immune system and to induce CTL responses, together with the lack of pre-existing anti-BV immunity in humans [14], its inherent inability to replicate in mammal hosts and the low cytotoxicity, make BV capsid display an appropriate candidate for the development of a vaccine vector.

## Materials and Methods

### Mice, cells and CpG-ODN

Six to eight week-old female C57BL/6 mice (H-2^b^) were obtained from Fundación Facultad de Ciencias Veterinarias (UNLP, La Plata, Argentina). C57BL/10 ScCr (TLR4-deficient) mice were generously provided by Dr. M. Maccioni (CIBICI-CONICET, Córdoba, Argentina). OT-I ones, expressing a transgenic T cell receptor designed to recognize ovalbumin residues 257–264 in the context of H-2K^b^ [53], were kindly provided by Dr. F.A. Goldbaum (Fundación Instituto Leloir, Buenos Aires, Argentina) and bred in our animal facility. They were maintained in our animal facilities, which met the terms of the Guide to the Care and Use of Experimental Animals, published by the Canadian Council on Animal Care (with the assurance number A5802-01 being assigned by the Office of Laboratory Animal Welfare (NIH)). All animals were maintained under specific pathogen-free conditions.

B3Z, a CD8 T cell hybridoma specific for OVA_257-264_ epitope in the context of K^b^ [54], was a generous gift from Dr. N. Shastri (University of California, Berkeley, CA). Murine B16 melanoma cell line was obtained from ATCC. MO5, a melanoma cell line expressing OVA [55], was a kind gift from Dr. Claude Leclerc (Institut Pasteur, Paris, France).

The CpG-ODN used was 1826 (TCCATGACGTTCCTGACGTT) (Operon Technologies, Alameda, CA, USA) and was complexed with the liposomal transfection reagent N-[1-(2,3-Dioleoyloxy)propyl]-N,N,Ntrimethylammonium methylsulfate (DOTAP, Roche Diagnostics Corporation, Indianapolis, IN). The optimal ratio of the CpG-ODN to DOTAP for complex formation was determined by dose titration (data not shown).

### Ethics Statement

This study was carried out in strict accordance with the recommendations in the Guide for the Care and Use of Laboratory Animals of the National Institutes of Health. Our Institutional Experimentation Animal Committee (authorization # 15-07-62010 and HCD resolution 450/07) approved the animal handling and experimental procedures. All experimental procedures were performed under isofluorane anesthesia. Sacrifice was carried out by CO_2_ inhalation. All efforts were made to minimize mice suffering.

### Capsid display vector

To construct a general BV vector for capsid display, the region corresponding to nt 468–1509 of the vp39 gene (GenBank Accession No. M22978) was amplified from purified DNA of AcMNPV by PCR using 5′ATCTAGA ***GGAGGTGGGGGATCG***GCGCTAGTGCCCGTGGGT3′ (specific sequence for nt 468-486 of vp39 gene, flexi peptide in bold and XbaI site underlined, without start codon) as the forward primer and 5′ AAAGCTT **TTA**GACGGCTATTCCTCC3′ (specific sequence for nt 1491–1509 of vp39 gene, stop codon in bold, HindIII sites underlined) as the reverse primer. The amplified fragment was digested with XbaI and HindIII and cloned into XbaI/HindIII-digested pFastBAC1 vector (Invitrogen), with the resulting plasmid being named pFBcap. The nucleotide sequence was confirmed by DNA sequencing, and the cDNAs encoding OVA and EGFP were amplified using specific primers to allow in frame fusions to the N-terminus of the VP39 protein. Forward primer 5′ TTCTAGAGT**ATG**GTGAGCAAGGGC3′ (specific sequence for nt 1–15 of gfp, start codon in bold, XbaI site underlined) and reverse primer 5′TTCTAGACTTGTACAGCTCGTC3′ (specific sequence for nt 705–717 of gfp, XbaI site underlined), forward primer 5′ AGGATCCAAT**ATG**CCTTTCAGAGTGACT3′ (specific sequence for nt 653–671 of ova, start codon in bold, BamHI site underlined) and reverse primer 5′ATCTAGAAGGGGAAACACATCTGCC3′ (specific sequence for nt 1206–1223 of ova, XbaI site underlined) were used. The amplified fragments were cloned into the XbaI (gfp) or XbaI/BamHI (ova) sites of the XbaI-pFBcap or XbaI/BamHI-pFBcap. The resulting plasmids were named pFBGFPcap or pFBOVAcap. Recombinant pFBcap constructs were individually transformed into *E.coli* DH10Bac cells (Invitrogen) to generate the corresponding recombinant bacmids, as suggested by the manufacturers. *Spodoptera frugiperda* 9 insect cells (Sf9, Invitrogen) were transfected with the recombinant bacmid DNA with Cellfectin (Invitrogen), and the recombinant BV vectors were amplified by repeated passages. Recombinant BV-OVAsur carrying the same OVA sequence than in BV-OVA but as a fusion protein to GP64 to allow surface display, was constructed as previously described [12].

### Insect cell culture and virus amplification and purification

AcMNPV virus was obtained from BaculoGold (Becton Dickinson Argentina S.R.L., Buenos Aires, Argentina). Briefly, BV were propagated in Sf9 cells in SF900 medium culture (2% FCS, 27°C). Then, the supernatants were harvested and cell debris removed by centrifugation (4,000× g, 15 min, 8°C). Infectious virus titers were calculated by end point dilution assay and converted to PFU/ml as described elsewhere [19]. Viruses were concentrated by centrifugation onto 25% sucrose cushion at 57,000× g (60 min, 8°C) in PBS. Virus stocks were considered free of endotoxin (<0.01 endotoxin U/ml) using the Limulus amebocyte lysate test, (E-TOXATE, Sigma). The amount of OVA incorporated per viral particle was estimated by the densitometry of bands in a western blot of serial dilutions of a known number of purified baculovirus particles and by comparing this with known amounts of ultrapure OVA protein, in a way very similar to that used by Kukkonen et al. [17]. The number of viral particles was estimated by FACS, as previously described [56]. Viral suspensions were routinely prepared from low passages and checked for the presence and quantity of OVA associated to viral particles. Recombinant BV yielded viral titers similar to BV-WT, suggesting that the viral cycle was not significantly affected by the presence of an additional capsid protein. The viral progeny exhibited similar viral titers and quantities of OVA associated to the nucleocapsid for at least five passages.

### Electron microscopy

For immunoelectron microscopy, BV-OVA particles treated with 1% Triton X-100 (Sigma) were bound to formvar-coated nickel grids, before being incubated with 0.1% BSA/PBS followed by an anti-OVA antibody. Grids were then incubated with a second antibody conjugated to gold particles (10 nm in diameter) and stained with 2% uranyl acetate. Samples were examined using a JEOL - SVC electron microscope.

### Flow Cytometry

Cells were labeled using standard procedures [57]. The following mAbs were used: anti-CD3ε (145-2C11 clone), anti-CD4 (L3T4, GK 1.5 or RM4-5 clone), anti-CD8α (Ly-2, 53-6.7 clone), anti-CD11c (HL-3 clone), anti-CD86 (B7.2, PO3.1 clone), anti-CD40 (HM40-3 clone), anti-MHC II (I-A/I-E, M5/114.15.2 clone) and anti-CD49b (DX5 clone), all purchased from BD Biosciences or eBioscience. Intracellular IFN-γ was measured by using the BD Cytofix/Cytoperm™ Plus Kit with GolgiStop, following the manufacturer's protocol. Cells were stained with anti-CD4-PE or anti-CD8-PE, and then with anti-IFN-γ-APC antibody (clone XMG1.2). Cells were either acquired in FACSCalibur or in FACSCanto II flow cytometers and analyzed using CellQuest (BD Biosciences) or FlowJo (Tree Star, Inc., Ashland, OR) software. In all cases, appropriate isotype controls were included.

### Antibodies and cytokine detection assays

Specific antibodies against OVA were determined by ELISA as detailed in Maletto et al. [58]. The levels of IL-6, IL-12 p40 and IFN-γ were measured in culture supernatants and serum samples by sandwich ELISA, following instructions from the manufacturers (BD Biosciences). The following antibodies were used for capture and detection, respectively: MP5-20F3 and MP-32C11 clones for anti-IL-6, C15.6 and C17.8 for anti-IL-12 p40 and XMG1.2 clones for anti-IFN-γ.

### Generation of Bone Marrow-derived Dendritic Cells (BMDC)

BMDCs were prepared according to Inaba et al. [59], with a few modifications. Briefly, bone marrow cells were collected from the femurs and tibias and then cultured (2.5×10^5^/ml) in 60 mm plates containing complete RPMI medium supplemented with 7.5% of GM-CSF from supernatant of the stably transfected GM-CSF-J558 cell line. Additional GM-CSF supernatant was added on days 3 and 7 of culture. Then, non-adherent and loosely-adherent BMDC were used at day 8 (>90% of the harvested cells expressed CD11c).

### DC purification

DCs were purified from spleen as reported Morón et al. [57]. Briefly, spleens were removed and treated with 0.4 U/ml collagenase and 100 U/ml DNase I (both from Roche Diagnostics). After inhibition of collagenase, the cell suspension was mixed with colloidal super-paramagnetic microbeads, conjugated to anti-CD11c mAb (magnetic-activated cell sorting [MACS]–anti-CD11c, N418 clone; Miltenyi Biotec). CD11c+ cells were positively selected with LS columns (Miltenyi Biotec). In some experiments, enriched DC populations were then purified by flow cytometry on a FACSAria II by co-labeling spleen cells with MACS and fluorescent anti-CD11c antibodies, as explained elsewhere [57].

### Antigen presentation assays

Splenic DCs from C57BL/6 mice (2×10^5^/well) were co-cultured with B3Z (10^5^/well cells) and recombinant BV-OVA or BV-GFP for 18 hours in 96-well culture microplates in a final volume of 0.2 ml of RPMI 1640, 2 mM GlutaMAX-I (all from Invitrogen), 5×10^−5^ M 2-ME (Sigma), 100 IU/ml penicillin, 100 g/ml streptomycin (both from PAA laboratories GmbH, Pasching, Germany), and 10% FCS (RPMI 10%). The activation of B3Z T cell hybridoma was monitored by testing the β-galactosidase activity using a colorimetric reaction with 0.15 mM chlorophenolred-β-D-galactopyranoside (CPRG, Roche Diagnostic Corporation), 100 mM 2-ME, 9 mM MgCl_2_, 0.125% IG-PAL CA 630 (Sigma) in PBS and measuring the OD at 595 nm.

### T-cell proliferation

T-cell proliferation was performed using OT-I T cells co-cultured with splenic DCs incubated with BV-OVA or BV-GFP. Splenic T cells from OT-I mice were purified by flow cytometry on a FACSAria II by labeling with anti-CD8β-PE antibody (53-5.8 clone, BD Biosciences). After sorting, OT-I cells were stained with 2.5 µM carboxyfluorescein diacetate, succinimidyl ester (CFSE) 2.5% FCS PBS and then washed extensively. Purified splenic DCs were incubated in the presence of BV at 37°C for 2 hours in a final volume of 0.2 ml of RPMI 10% FCS and then washed. OT-I cells were added to DCs and cultured for 72 hours. Then, cultured cells were harvested, labeled with anti-CD8, anti-CD3 and anti-CD25 antibodies and 7AAD to exclude dead cells, and analyzed by flow cytometry. Proliferation was determined by the dilution of CFSE content in CD3+ CD8 7AAD- cells.

### *In vivo* killing assay

Naïve syngenic splenocytes were pulsed with 10 µg/ml OVA_257–264_ peptide (SIINFEKL) (30 min, 37°C), washed extensively and labeled with a high concentration (3 µM) of CFSE (Invitrogen). A non-pulsed control population was labeled with a low concentration (0.5 µM) of CFSE. Then, CFSE^high^- and CFSE^low^-labeled cells were mixed at a 1∶1 ratio (1×10^7^ cells of each population) and injected i.v. into immunized mice. The number of CFSE+ cells remaining in the spleen after 20 hours was determined by flow cytometry. Cytotocixity was expressed as percentage of lysis, calculated from [1-(r_immune_/r_control_)]x100, where r is given by the expression of%CFSE^low^/%CFSE^high^ cells for immune and non-immunized (control) mice, respectively.

### *In vivo* tumor protection experiment

Prophylactic vaccination: Groups of eight C57BL/6 mice were immunized by a single i.v. injection of 5×10^7^ PFU BV-WT, BV-OVA or PBS. Seven days later, immunized mice were challenged with an s.c. injection of 1×10^5^ MO5 cells. Therapeutic vaccination: Groups of eight C57BL/6 mice were s.c. injected with 1×10^5^ MO5 cells. Mice showing palpable tumors were then injected with PBS, BV-WT or BV-OVA as follows: days 7 (i.v., 5×10^7^ PFU), 11 and 17 (s.c. near tumor, 1×10^7^ PFU) and day 21 (i.t. 1×10^7^ PFU). Mice were monitored periodically, recording tumor volume and survival. Mice with tumors longer than 2.5 cm were sacrificed. The tumor volume was calculated from (d2 × D)/2, where D =  longest diameter and d =  diameter perpendicular to D.

### Statistics

Statistical analysis was performed using the Bonferroni post test, one way ANOVA analysis, Student's *t* test and comparison of survival curves was done using Logrank test with GraphPad Prism (La Jolla, CA). Values of p<0.05 were considered significant.
